# Using adult care visits to diagnose HIV infection in children, Burkina Faso

**DOI:** 10.2471/BLT.23.289606

**Published:** 2024-01-29

**Authors:** Souleymane Tassembédo, Isidore Tiandiogo Traoré, Makoura Traoré-Barro, Ismael Diallo, Daouda Maré, Fatimata Diallo-Barry, Camille Rajaonarivelo, Bethem Coulibaly, Amélie Nikiema, Armel Poda, Philippe Vande Perre, Nicolas Nagot

**Affiliations:** a Centre Muraz, Institut National de Santé Publique, Bobo-Dioulassso, Programme de Recherche sur les Maladies Infectieuses, Centre Muraz 2054 Avenue Mamadou Konate, Bobo-Dioulasso, Burkina Faso.; b Institut Supérieur des Sciences de la Santé, Université Nazi Boni, Bobo-Dioulasso, Burkina Faso.; c Département de Médecine, Centre Hospitalier Universitaire Yalgado Ouedraogo, Ouagadougou, Burkina Faso.; d Revs Plus, Bobo-Dioulasso, Burkina Faso.; e Centre Médical avec Antenne Chirurgicale de Pissy, Direction Régionale de la Santé du Centre, Ouagadougou, Burkina Faso.; f Centre Oasis, Association Africain Solidarité, Ouagadougou, Burkina Faso.; g Centre Médical avec Antenne Chirurgicale de Dafra, Direction Régionale de la Santé des Hauts-Bassins, Bobo-Dioulasso, Burkina Faso.; h Centre Médical avec Antenne Chirurgicale de Do, Direction Régionale de la Santé des Hauts-Bassins, Bobo-Dioulasso, Burkina Faso.; i Département de Médecine, Centre Hospitalier Universitaire Souro Sanou, Bobo-Dioulasso, Burkina Faso.; j Pathogenesis and Control of Chronic and Emerging Infections, Montpellier University, Institut national de la santé et de la recherche médicale (INSERM), Montpellier, France.

## Abstract

**Objective:**

To estimate the feasibility, positivity rate and cost of offering child testing for human immunodeficiency virus (HIV) to mothers living with HIV attending outpatient clinics in Burkina Faso.

**Methods:**

We conducted this implementation study in nine outpatient clinics between October 2021 and June 2022. We identified all women ≤ 45 years who were attending these clinics for their routine HIV care and who had at least one living child aged between 18 months and 5 years whose HIV status was not known. We offered these mothers an HIV test for their child at their next outpatient visit. We calculated intervention uptake, HIV positivity rate and costs.

**Findings:**

Of 799 eligible children, we tested 663 (83.0%) and identified 16 new HIV infections: 2.5% (95% confidence interval, CI: 1.5–4.1). Compared with HIV-negative children, significantly more HIV-infected children were breastfed beyond 12 months (*P*-value: 0.003) and they had not been tested before (*P*-value: 0.003). A significantly greater proportion of mothers of HIV-infected children were unaware of the availability of child testing at 18 months (*P*-value: < 0.001) and had more recently learnt their HIV status (*P*-value: 0.01) than mothers of HIV-negative children. The intervention cost 98.1 United States dollars for one child testing HIV-positive. Barriers to implementing this strategy included shortages of HIV tests, increased workload for health-care workers and difficulty accessing children not living with their mothers.

**Conclusion:**

Testing HIV-exposed children through their mothers in outpatient clinics is feasible and effective in a low HIV-prevalence setting such as Burkina Faso. Implementation of this strategy to detect undiagnosed HIV-infected children is recommended.

## Résumé

**Objectif:**

Évaluer la faisabilité, le taux de positivité et le coût que représenterait un dépistage du virus de l'immunodéficience humaine (VIH) chez les enfants s'il est proposé aux mères vivant avec le VIH qui se rendent dans des cliniques externes au Burkina Faso.

**Méthodes:**

Nous avons déployé cette étude de mise en œuvre dans neuf cliniques externes entre octobre 2021 et juin 2022. Nous avons identifié toutes les femmes âgées de ≤ 45 ans qui fréquentaient ces cliniques pour leurs soins de routine dans le cadre d'une prise en charge du VIH et avaient au moins un enfant vivant de minimum 18 mois et maximum 5 ans, dont le statut VIH n'était pas connu. Nous avons proposé à ces mères de procéder au dépistage de leur enfant lors de leur prochaine visite à la clinique. Enfin, nous avons calculé le nombre de recours à cette intervention, le taux de positivité du VIH et les coûts.

**Résultats:**

Sur 799 enfants éligibles, nous en avons testé 663 (83,0%) et avons détecté 16 nouvelles infections au VIH: 2,5% (intervalle de confiance de 95%: 1,5–4,1). Par rapport aux enfants séronégatifs, un nombre nettement supérieur d'enfants séropositifs avaient été allaités au-delà de 12 mois (valeur-*p*: 0,003) et n'avaient pas été testés auparavant (valeur-*p*: 0,003). Un pourcentage sensiblement plus élevé de mères d'enfants séropositifs ignoraient l'existence d'un dépistage chez les enfants âgés de 18 mois (valeur-*p*: < 0,001) et avaient appris leur statut VIH plus récemment (valeur-*p*: 0,01) que les mères d'enfants séronégatifs. Cette intervention a coûté 98,1 dollars américains par enfant séropositif. Parmi les obstacles à cette stratégie figurent la pénurie de tests de dépistage du VIH, l'augmentation de la charge de travail pour les professionnels de la santé et les difficultés d'accès aux enfants qui ne vivent pas avec leur mère.

**Conclusion:**

Tester les enfants exposés au VIH par l'intermédiaire de leur mère dans les cliniques externes est faisable et efficace dans les lieux où la prévalence du VIH est faible, comme au Burkina Faso. Nous recommandons l'application de cette stratégie pour identifier les enfants séropositifs non diagnostiqués.

## Resumen

**Objetivo:**

Estimar la viabilidad, la tasa de positividad y el coste de ofrecer pruebas de detección del virus de la inmunodeficiencia humana (VIH) en niños a las madres seropositivas que acuden a las clínicas de atención ambulatoria de Burkina Faso.

**Métodos:**

Se realizó este estudio de implementación en nueve clínicas de atención ambulatoria entre octubre de 2021 y junio de 2022. Se identificó a todas las mujeres ≤45 años que acudían a estas clínicas para su atención de rutina del VIH y que tenían al menos un hijo vivo de entre 18 meses y 5 años, cuyo estado serológico respecto al VIH se desconocía. Se ofreció a estas madres una prueba del VIH para su hijo en su siguiente consulta externa. Se calcularon la aceptación de la intervención, la tasa de positividad del VIH y los costes.

**Resultados:**

De 799 niños que cumplían los requisitos, se realizaron pruebas a 663 (83,0%) y se identificaron 16 nuevas infecciones por VIH: 2,5% (intervalo de confianza del 95%: 1,5-4,1). En comparación con los niños seronegativos, un número significativamente mayor de niños infectados por el VIH fueron amamantados más de 12 meses (valor de *p*: 0,003) y no se habían sometido a pruebas anteriormente (valor de *p*: 0,003). Un porcentaje significativamente mayor de madres de niños infectados por el VIH desconocía la disponibilidad de pruebas infantiles a los 18 meses (valor de *p*: <0,001) y había conocido más recientemente su estado serológico respecto al VIH (valor de *p*: 0,01) que las madres de niños seronegativos. La intervención costó US$ 98,1 por niño seropositivo. Entre los obstáculos a la aplicación de esta estrategia se encontraban la escasez de pruebas del VIH, el aumento de la carga de trabajo de los profesionales sanitarios y la dificultad para acceder a los niños que no vivían con sus madres.

**Conclusión:**

La realización de pruebas a niños expuestos al VIH a través de sus madres en clínicas de atención ambulatoria es viable y eficaz en un entorno de baja prevalencia del VIH como Burkina Faso. Se recomienda la aplicación de esta estrategia para detectar a los niños infectados por el VIH no diagnosticados.

## ملخص

**الغرض:**

تقدير مدى جدوى، ومعدل الإصابة، وتكلفة عرض إجراء اختبار للكشف عن الإصابة بفيروس نقص المناعة البشرية (HIV)، على الأطفال المصاحبين للأمهات المصابات بفيروس نقص المناعة البشرية المترددات على العيادات الخارجية في بوركينا فاصو.

**الطريقة:**

أجرينا هذه الدراسة التنفيذية في تسع عيادات خارجية في الفترة ما بين أكتوبر/تشرين أول 2021 ويونيو/حزيران 2022. وقد حددنا جميع السيدات اللاتي تكون أعمارهن 45 عامًا أو أقل، واللاتي كن يترددن على هذه العيادات للحصول على الرعاية الروتينية لفيروس نقص المناعة البشرية، واللاتي كان لديهن طفل واحد على الأقل على قيد الحياة، يتراوح عمره بين 18 شهرًا و5 سنوات، ومن غير المعروف حالة إصابته بفيروس نقص المناعة البشرية من عدمها. لقد عرضنا على هؤلاء الأمهات إجراء اختبار فيروس نقص المناعة البشرية لأطفالهن في زيارتهن التالية للعيادة الخارجية. قمنا باحتساب استيعاب التدخل، ومعدل الإصابة بفيروس نقص المناعة البشرية، والتكاليف.

**النتائج:**

من بين 799 طفلاً مؤهلاً، قمنا باختبار 663 (%83.0) منهم، واكتشفنا 16 إصابة جديدة بفيروس نقص المناعة البشرية: %2.5 (بفاصل ثقة مقداره %95: %1.5 إلى %4.1). بالمقارنة مع الأطفال غير المصابين بفيروس نقص المناعة البشرية، فإن عدد أكبر بكثير من الأطفال المصابين بفيروس نقص المناعة البشرية تم إرضاعهم طبيعيًا لما يزيد عن 12 شهرًا (نسبة الاحتمال:  0.003)، ولم يتم اختبارهم من قبل (نسبة الاحتمال: 0.003). كانت نسبة أكبر بشكل ملحوظ من أمهات الأطفال المصابين بفيروس نقص المناعة البشرية، على غير دراية بإتاحة اختبارات الأطفال عند عمر 18 شهرًا (نسبة الاحتمال: أقل من 0.001)، وقد أصبحن مؤخرًا على دراية أكبر بحالة فيروس نقص المناعة البشرية لديهن (نسبة الاحتمال: 0.01) مقارنة بأمهات الأطفال الذين تبين عدم إصابتهم بفيروس نقص المناعة البشرية. بلغت تكلفة التدخل 98.1 دولارًا أمريكيًا لكل طفل ثبتت إصابته بفيروس نقص المناعة البشرية. وشملت العوائق التي تحول دون تنفيذ هذه الاستراتيجية: النقص في اختبارات فيروس نقص المناعة البشرية، وزيادة عبء العمل على العاملين في مجال الرعاية الصحية، وصعوبة الوصول إلى الأطفال الذين لا يعيشون مع أمهاتهم.

**الاستنتاج:**

إن اختبار الأطفال المعرضين للإصابة بفيروس نقص المناعة البشرية من خلال أمهاتهم في العيادات الخارجية، أمر ممكن وفعال في البيئات ذات الانتشار المنخفض لفيروس نقص المناعة البشرية مثل بوركينا فاصو. يُوصى بتنفيذ هذه الاستراتيجية للكشف عن الأطفال المصابين بعدوى نقص المناعة البشرية، ولم يتم تشخيصهم بعد.

## 摘要

**目的:**

评估向到布基纳法索门诊就诊的感染 HIV 的母亲提供为其孩子进行人类免疫缺陷病毒 (HIV) 检测的可行性、诊断阳性率和费用情况。

**方法:**

在 2021 年 10 月至 2022 年 6 月期间，我们在 9 家门诊部开展了这项实施研究。我们确定了到这些门诊部接受常规的 HIV 护理并且至少有一个年龄在 18 个月至 5 岁之间的存活的孩子，且孩子的 HIV 状况未知的所有年龄为 45 岁及以下的女性。我们为这些母亲提供了在下次到门诊就诊时为其孩子进行 HIV 检测的机会。我们计算了干预措施的接受率、HIV 阳性率和费用。

**结果:**

在 799 名符合条件的孩子中，我们检测了 663 名 (83.0%) ，并确定了 16 名新的 HIV 感染者：2.5%（95% 置信区间：1.5-4.1）。与 HIV 呈阴性的孩子相比，感染 HIV 的孩子中母乳喂养超过 12 个月的明显偏多（*P* 值：0.003），并且他们之前没有接受过该检测（*P* 值：0.003）。与 HIV 呈阴性孩子的母亲相比，感染 HIV 的孩子的母亲中不知道在其 18 个月时可以进行儿童检测（*P* 值：< 0.001）的明显占更大的比例，并且这些母亲中最近才知道自己 HIV 状况（*P* 值：0.01）的所占比例也高一些。对一名 HIV 检测呈阳性的孩子采取干预措施的费用为 98.1 美元。实施这一策略的障碍包括缺乏 HIV 检测、卫生保健工作者的工作量增加以及难以接触未与母亲同住的孩子。

**结论:**

在 HIV 流行率较低的国家/地区（如布基纳法索），通过母亲在门诊就诊对暴露于 HIV 的孩子进行检测是可行且有效的。建议实施这一策略，以发现未确诊的感染 HIV 的孩子。

## Резюме

**Цель:**

Оценить целесообразность, процент лиц с положительной реакцией и стоимость проведения тестирования детей на выявление вируса иммунодефицита человека (ВИЧ) для матерей, живущих с ВИЧ и посещающих амбулаторные клиники в Буркина-Фасо.

**Методы:**

Данное исследование проводилось в девяти амбулаторных клиниках в период с октября 2021 года по июнь 2022 года. Были выявлены все женщины в возрасте до 45 лет, которые посещали эти клиники для планового лечения ВИЧ-инфекции и у которых был хотя бы один живой ребенок в возрасте от 18 месяцев до 5 лет, чей ВИЧ-статус не был известен. Этим матерям было предложено пройти тест на ВИЧ для их ребенка в ходе следующего амбулаторного приема. Были рассчитаны показатели охвата процедурами, процент лиц с положительной реакцией на ВИЧ и затраты.

**Результаты:**

Из 799 соответствующих критериям детей обследовано 663 (83,0%), выявлено 16 новых случаев ВИЧ-инфекции: 2,5% (95%-й ДИ: 1,5–4,1). По сравнению с детьми с отрицательным ВИЧ-статусом значительно большая часть ВИЧ-инфицированных детей находилась на грудном вскармливании до 12 месяцев (*P*-значение: 0,003) и не проходила тестирование ранее (*P*-значение: 0,003). Значительно большая доля матерей ВИЧ-инфицированных детей не знали о доступности тестирования детей в возрасте 18 месяцев (*P*-значение: < 0,001) и узнали о своем ВИЧ-статусе недавно (*P*-значение: 0,01) по сравнению с матерями детей с отрицательным ВИЧ-статусом. Стоимость процедуры составила 98,1 доллара США на одного ребенка с положительным ВИЧ-статусом. Препятствиями для реализации этой стратегии стали нехватка тестов на ВИЧ, повышенная нагрузка на медицинский персонал и сложности с доступом к детям, не проживающим со своими матерями.

**Вывод:**

Тестирование ВИЧ-инфицированных детей через их матерей в амбулаторных клиниках возможно и эффективно в регионах с низкой распространенностью ВИЧ, таких как Буркина-Фасо. Рекомендуется внедрение этой стратегии для выявления недиагностированных ВИЧ-инфицированных детей.

## Introduction

Despite great improvements in the response to human immunodeficiency virus (HIV), the 90–90–90 targets of the Joint United Nations Programme on HIV and AIDS (acquired immunodeficiency syndrome) for 2020 were not met in most countries in sub-Saharan Africa. For the first of these targets – by 2020, 90% of all people living with HIV will know their HIV status – an estimated 90% (95% confidence interval, CI: 73–98) of adults ≥ 15 years infected with HIV knew their status in southern and eastern Africa; this figure was 81% (95% CI: 67–98) in western and central Africa.^1^ These figures fall to 65% (95% CI: 46–80) and 35% (95% CI: 26–47), respectively, for children aged 0–14 years.^2^ This large gap between children and adults highlights important failures in HIV testing during the period of HIV exposure in utero to the end of breastfeeding.

The World Health Organization (WHO) recommends that infants exposed to HIV have nucleic acid testing by 4–8 weeks of age (early infant diagnosis), with a final diagnosis made at 18 months or 3 months after breastfeeding cessation, whichever is the most appropriate.^3^ Testing for early infant diagnosis is low, especially in western and central Africa where only 33% (95% CI: 25–47) of infants exposed to HIV are diagnosed by 8 weeks.^4^ Poor access to molecular testing and shortages of tests or reagents are common reasons for this situation.^5^ The final diagnosis is crucial and is often the last testing opportunity for HIV-exposed children. Although the 18-month testing can be done with a simple serological test, compliance is still low in sub-Saharan Africa, around 50% overall.^6^^,^^7^ Without diagnosis and treatment, an estimated 52.5% of HIV-infected children will die by the age of 2 years.^8^

Studies in sub-Saharan Africa document low rates of HIV testing in children aged 2 years and older.^9^^–^^11^ Factors associated with poor testing uptake include loss to follow-up of mothers or infants from health facilities, shortages in diagnostic reagents and delayed results.^5^

Provider-initiated testing and counselling and index family testing can help increase diagnosis of HIV in infants.^12^^,^^13^ Index family testing in high-prevalence settings in priority countries of the *Global plan towards the elimination of new HIV infections among children by 2015 and keeping their mothers alive*^11^^,^^14^^–^^18^ has relied mainly on fathers as the index case and targeted mostly adolescents. In Kenya, 2848 children aged 0–14 years whose mothers and fathers were newly enrolled in HIV care were tested, 127 (4.5%) of whom were newly diagnosed with HIV.^19^

Few studies on testing children through their index parents have been conducted in west and central Africa.^1^ In Cameroon, mothers and fathers attending clinics for antiretroviral therapy (ART) were offered testing for their children aged 0–15 years.^10^ Only 46.2% (571/1236) of the parents had at least one child tested, with better acceptance when the mother was the index case. In Nigeria, parents contacted at various entry points were offered HIV testing for their children aged 0–15 years. Testing uptake reached 83.6% (1822/2180), and 3.5% (24/683) of the children were HIV-positive.^11^

In west Africa, HIV is more prevalent in women and they have better access to screening and treatment. Women are also more adherent to follow-up than men,^20^^–^^23^ and children from index mothers are tested more frequently.^10^ Consequently, a case-finding strategy with women as the index case may be more appropriate in west Africa.

In this study, we estimated the feasibility, HIV positivity rate and cost of a strategy to test children for HIV in Burkina Faso by targeting women of childbearing age who were routinely followed up in outpatient clinics as index cases. 

## Methods

### Design and setting

We used an effectiveness–implementation hybrid trial type 1 study design.^24^

Burkina Faso has a low HIV prevalence estimated at 0.7% (95% CI: 0.5–0.8) in adults aged 15–49 years.^1^ The mother to child transmission (MTCT) rate, including through breastfeeding, was estimated at 12% (95% CI: 9.2–16.1) in 2020.^1^ Coverage of early infant diagnosis estimated from national programme data is 10% (95% CI: 8.4–12.8);^1^ no data are available on the 18-month HIV test coverage. The prevention of mother to child transmission (PMTCT) programme covers care for HIV-infected mothers and infants at maternal and child units at *Centres de Santé et de Promotion Sociale* (lowest level of health-care services) until the child is 2 years. After this age, the child exits the PMTCT programme and is referred to a paediatric HIV clinic for care.

We implemented our strategy in the two cities of Burkina Faso, Ouagadougou and Bobo-Dioulasso, between October 2021 and June 2022. We recruited participants from nine public outpatient clinics at two tertiary hospitals (*Centre Hospitalo-Universitaire Yalgado Ouedraogo* and *Centre Hospitalo-Universitaire Souro Sanou*), three district hospitals (Do, Dafra and Boulmiougou) and four community clinics (*Centre Oasis*, *Clinique Yerelon Ouaga*, *Clinique Yerelon Bobo-Dioulasso* and *Centre REVS Plus Bobo-Dioulasso*). We chose these clinics to match the local HIV care situation and include all levels of the health system. The usual staff at these clinics implemented the strategy. When we started this study, HIV clinics had no testing capacity because their mandate was only to treat people referred with a confirmed HIV diagnosis. The National AIDS Control Programme provided testing kits, and we trained staff in their use for children.

### Population and sample size

The study population was women living with HIV followed at the outpatient clinics, and their biological children aged 18 months to 5 years whose HIV status was unknown. In this study, female sex was assessed by the individual’s self-identification as a mother. No attempt to assess gender was made.

Based on a 2% positivity rate in exposed children older than 18 months in Burkina Faso,^25^ a 10% non-response rate, 1% precision and a 5% uncertainty level, the sample size needed was 753 children.

### Implementation

We identified all women followed at the nine sites. We then contacted the women aged 45 years or younger by telephone or at the clinic to determine whether they had at least one living child aged between 18 months and 5 years whose HIV status was not known. We offered mothers with at least one eligible child an HIV diagnosis for their child, and asked them to bring the child at their next usual outpatient visit, or at an earlier time if they preferred. Additionally, we conducted twice-weekly counselling sessions on postnatal MTCT of HIV and its prevention to educate women and encourage child testing. We excluded women without a child in this age group or who had a child whose HIV status was known.

After the mother gave her consent to participate, the clinic nurse recorded the mother’s sociodemographic characteristics, HIV testing history of her child, and infant feeding history using a standardized questionnaire on a tablet.

All eligible children underwent HIV serological screening according to the national protocol. Clinic staff tested the children using a rapid test (Alere Determine HIV-1/2 Ab, Abbott, Chiba, Japan). If the test was negative, the child was considered HIV-negative. If the test was positive, staff did a second test (OnSite^®^ HIV 1/2 Ab Plus Combo Rapid Test, CTK Biotech, Poway, United States of America) to confirm HIV infection and determine the type (HIV-1 or HIV-2 or dual reactivity). For indeterminate results, clinic staff offered repeat testing 1 month later or a nucleic acid test whenever possible. Children who were still breastfeeding at the time of the study who tested negative at the first visit were assessed again at the next routine visit until 3 months after breastfeeding cessation.

The staff and mothers received no financial compensation, as the child testing was during a routine maternal visit for HIV care and took little time.

### Outcomes

We determined feasibility of the strategy by assessing the difficulties encountered by HIV services in implementing the strategy and the uptake of the intervention by the mothers. We measured uptake as the proportion of mothers who brought their children for testing of the total number of mothers contacted.

We estimated the HIV positivity rate as the proportion of children who tested HIV-positive of all children tested.

We evaluated the acceptability of the strategy during on-site monitoring visits, by asking health-care workers to provide feedback on the challenges encountered in implementing the strategy.

To estimate the financial costs of the strategy, we used data from the National AIDS Control Programme. We categorized costs as capital or recurrent. Recurrent costs included supplies (testing kits, gloves, alcohol, swabs and telephone calls to mothers) and building operation and maintenance. Capital costs included start-up training, and sensitization of mothers through posters advocating HIV testing of exposed infants. We considered building operation and maintenance costs to be negligible. We priced supplies according to central pharmacy price lists. Sensitization costs were the price of poster printing. We estimated costs in 2021–2022 Financial Community of Africa (CFA) francs and converted to United States dollars (US$) using the period average exchange rate of 600 CFA francs = US$ 1. We only applied confirmatory testing costs to children testing positive for HIV. 

### Statistical analysis

We summarized the characteristics of the mothers and children as proportions with 95% binomial CIs and as medians and interquartile ranges (IQR) for categorical and continuous variables, respectively.

We compared the characteristics of the children found positive and negative for HIV using the Pearson χ^2^ test or Fisher exact test for categorical variables, and the Mann–Whitney U-test for continuous variables. 

We used Stata, version 16.0 (StataCorp, College Station, USA) for data analyses.

### Ethical considerations

The Burkina Faso Health Research Ethics Committee approved the study protocol. All women who agreed to participate signed an informed consent form. We referred HIV-infected children to an appropriate HIV paediatric care centre.

## Results

**Fig. 1:**
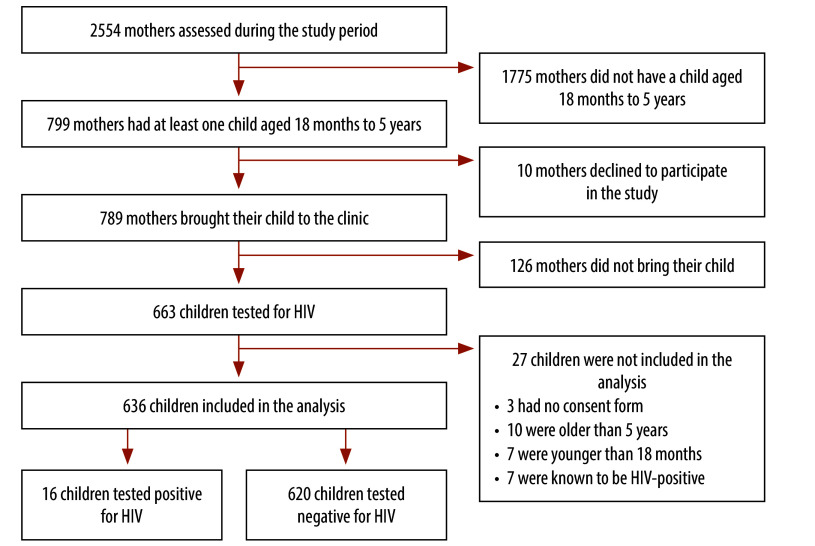
Flowchart of recruitment of women and children to diagnose HIV infection in children, Burkina Faso, 2021–2022

Between October 2021 and March 2022, we assessed 2554 women living with HIV for participation in the study. Of these women, 799 had eligible children and 789 agreed to participate (response rate 98.7%). Of the 789 mothers, 663 (84.0%) brought their children for HIV testing. We excluded 27 children from the analysis (Fig. 1). 

### Participant characteristics

The median maternal age of the mothers was 37 years (IQR: 32–41 years), and 43.1% (274/636) had no formal education. Most mothers (83.5%; 531/636) were living as a couple (with a spouse or partner) and 89.1% (567/636) said that they had disclosed their HIV status to their partner and/or family. The median age of the children was 3 years (IQR: 2–4 years) and 52.8% (336/636) were female. Breastfeeding had stopped after 12 months for 55.3% (332/600) of the children, and 41.3% (263/636) of the mothers reported that their child had tested negative for HIV before or at 12 months of age (Table 1).

**Table 1:** Characteristics of mothers and children, Burkina Faso, 2021–2022

Characteristic	Value (*n* = 636)
**Mothers**	
City of residence, no. (%)	
Ouagadougou	286 (45.0)
Bobo-Dioulasso	350 (55.0)
Location HIV care centre, no. (%)	
Tertiary hospital	134 (21.1)
District hospital	252 (39.6)
Community clinic	250 (39.3)
Age, in years, median (IQR)	37 (32–41)
Age range, in years, no. (%)	
≤ 25	47 (7.4)
> 25	589 (92.6)
Main occupation, no. (%)	
Informal sector worker	592 (93.1)
Formal sector worker	44 (6.9)
Education, no. (%)	
No formal education	274 (43.1)
Any formal education	362 (56.9)
Partner status, no. (%)	
Lives with a partner	531 (83.5)
Does not live with a partner	105 (16.5)
Disclosed HIV status,^a^ no. (%)	567 (89.1)
Reported intimate partner violence,^b^ no. (%)	161 (25.3)
Time since HIV diagnosis in years, median (IQR)	7 (4–13)
Time on ART in years, median (IQR)	7 (3–11)
Reported missed doses of ART,^c^ no. (%)	191 (30.0)
**Children**	
Age in years, median (IQR)	3 (2–4)
Age range, no. (%)	
18 months to 2 years	279 (43.9)
3 years	136 (21.4)
4 years	106 (16.7)
5 years	115 (18.1)
Sex, no. (%)	
Male	300 (47.2)
Female	336 (52.8)
Age at cessation of breastfeeding in months, median (IQR)	14 (12–18)
Age at cessation of breastfeeding in months,^d^ no. (%)	
≤ 12	268 (44.7)
> 12	332 (55.3)
Tested for HIV by 12 months of age, no. (%)	263 (41.3)
Mother unaware of the 18-month test, no. (%)	100 (15.7)

ART: antiretroviral therapy; HIV: human immunodeficiency virus; IQR: interquartile range.

^a^ To partner, family and friends.

^b^ Intimate partner violence refers to behaviour within an intimate relationship that causes physical, sexual, or psychological harm, including acts of physical aggression, sexual coercion, psychological abuse and controlling behaviour. This World Health Organization definition covers violence by current and former spouses and partners.^26^

^c^ Several days during pregnancy and/or breastfeeding.

^d^ Data were missing for 36 children.

### HIV-positive children

Among the 636 children exposed to HIV, 16 were HIV-positive, giving an HIV prevalence of 2.5% (95% CI: 1.5–4.1). The mothers of the HIV-positive children were significantly younger, and had been diagnosed with HIV and started treatment more recently than mothers of uninfected children. Compared with the HIV-negative children, a significantly greater proportion of HIV-positive children had been breastfed beyond 12 months (*P*-value: 0.003). Only 37.5% (6/16) of mothers of HIV-infected children were aware of systematic HIV testing at 18 months for their child compared with 85.5% (530/620) of other mothers (*P*-value: < 0.001). Additionally, a significantly greater proportion of mothers of HIV-infected children reported missing ART during pregnancy (*P*-value: 0.02; Table 2).

**Table 2:** Characteristics of the children by HIV status, Burkina Faso, 2021–2022

Characteristic	Children positive for HIV (*n* = 16)	Children negative for HIV (*n* = 620)	*P*
**Mothers**			
City, no. (%)			0.54
Ouagadougou	6 (37.5)	280 (45.2)	
Bobo-Dioulasso	10 (62.5)	340 (54.8)	
Mother age in years, median (IQR)	31.5 (26.5–36.0)	37.0 (32.0–41.0)	0.006
Main occupation, no. (%)			0.62
Informal sector workers	16 (100)	576 (92.9)	
Formal sector workers	0 (0)	44 (7.1)	
Education, no. (%)			0.96
No formal education	7 (43.8)	267 (43.1)	
Any formal education	9 (56.2)	353 (56.9)	
Partner status, no. (%)			0.10
Lives with a partner	11 (68.8)	520 (83.9)	
Does not live with a partner	5 (31.3)	100 (16.1)	
HIV status not disclosed, no. (%)	2 (12.5)	67 (10.8)	0.69
Unaware of the 18-month test, no. (%)	10 (62.5)	90 (14.5)	< 0.001
Reported intimate partner violence, no. (%)	4 (25.0)	157 (25.3)	0.98
Time since HIV diagnosis in years, median (IQR)	4.5 (1–9)	8 (4–13)	0.01
Time on ART in years, median (IQR)	1.5 (1–6.5)	7 (3–11)	0.005
Forgot to take ART during pregnancy, no. (%)	9 (56.3)	182 (29.4)	0.02
Other children known to be HIV-infected, no. (%)			0.06
1	11 (68.8)	281 (45.3)	
≥ 2	5 (31.2)	339 (54.7)	
**Children**			
Age in years, median (IQR)	2.5 (2–4)	3 (2–4)	0.88
Female sex	11 (68.8)	325 (52.4)	0.2
Breastfeeding cessation at > 12 months, no. (%)	14 (93.3)^a^	318 (54.4)^b^	0.003
No early infant diagnosis, no. (%)	15 (93.7)	358 (57.7)	0.003
No prior testing at 18 months, no. (%)	16 (100)	306 (49.3)	< 0.001

ART: antiretroviral therapy; HIV: human immunodeficiency virus; IQR: interquartile range.

^a^ Data were missing for one child.

^b^ Data were missing for 35 children.

Of the 16 HIV-infected children, 10 were started on ART on the same day as diagnosis, five started within 1 week and one started 1 month after the diagnosis.

### Feasibility of the intervention

The challenges of the intervention reported by the clinic staff included a shortage of HIV tests, high turnaround of health workers and increased workload as a result of the intervention. Some staff were also uncomfortable using tablets to record the participants’ data. The challenges reported by mothers were transportation costs to return with their child; the fact that their child was not living with them (living with their fathers, grandparents or other relatives); and their child was at school.

Recruitment and testing of children was slower at the tertiary hospital outpatient clinics than the community-based primary health care facilities.

### Cost of the intervention

The total intervention cost for the 636 children in this analysis was US$ 1569.7, with telephone costs to contact mothers accounting for the largest share (52.6%; US$ 825.0). The average costs were US$ 2.4 for testing one child, and US$ 98.1 per child testing positive for HIV (Table 3).

**Table 3:** Intervention costs and averages cost per child tested for HIV, Burkina Faso, 2021–2022

Item	Cost, US$	% of total cost
**Capital costs**		
Start-up training	120.0	7.6
Sensitization	150.0	9.6
Total capital costs	270.0	17.2
**Recurrent costs**		
Test kits (screening)	318.0	20.3
Test kits (confirmatory)	40.0	2.5
Supplies	116.7	7.4
Airtime	825.0	52.6
**Total recurrent costs**	1299.7	82.8
**Total intervention costs**	1569.7	NA
**Average cost per child tested^a^**	2.5	NA
**Average cost per child found to be living with HIV^b^**	98.1	NA

HIV: human immunodeficiency virus; NA: not applicable; US$: United States dollars.

^a^ Does not include the cost of confirmatory testing.

^b^ Includes the cost of confirmatory testing.

## Discussion

Our strategy of testing children of HIV-infected mothers routinely followed at outpatient clinics was efficient at identifying children with HIV whose status was unknown. Without this intervention, these children would either never have been diagnosed or would have been diagnosed later at the AIDS stage when the chance of survival is low. The intervention only identified children who had survived after being infected with HIV. With an estimated survival rate of 50% at 2 years and 20% at 5 years,^8^ at least two to three times as many children are likely to have died before we could diagnose them. This finding emphasizes the need for systematic identification of eligible children aged 18 months to 2 years in women attending HIV care centres. Efforts are also needed to identify undiagnosed mothers who become infected after delivery or breastfeeding.

Importantly, our real-life intervention was feasible and acceptable. The 98.7% acceptance rate of the mothers was higher than other studies with index parents including mothers: pooled estimate 51.7% (95% CI: 10.4–92.9) in sub-Saharan Africa;^27^ 90.7% (807/890) in Nigeria;^11^ and 93.5% (431/461) in Malawi.^18^ This finding suggests that an HIV medical consultation may avoid potential stigma reported in other contexts. However, 15.7% of our mothers did not bring their children for HIV testing. The reasons for this situation include: child no longer living with their mother; mother’s unwillingness to have their child tested; approval needed from the father whom the mother was reluctant to ask; father’s refusal to allow their child to be tested; lack of money for transportation; and remoteness of the health facility.^28^ The short study duration did not allow us to assess if some of these mothers could have overcome these difficulties and eventually had their child tested. 

The fact that district and tertiary hospitals had more difficulty recruiting and testing children than community-based clinics at the beginning of the study was expected, as the community-based clinics have extensive experience in HIV testing and counselling. Therefore, these clinics were better prepared to start screening children. District and tertiary hospital outpatient clinics focus on care not testing, particularly for children. Some staff found the use of tablets for data collection difficult. However, such data are not required for our strategy to be routinely adopted.

In our study, 83.0% of eligible children were tested, which is higher than a study in Kenya in which parents at outpatient clinics were given incentives to bring their child for testing.^15^ Child testing uptake was only 33.3% (30/90) for the group with no incentive, and 61.4% (54/88) for the group given the highest incentive (US$ 10).^15^ A study in Nigeria in which child testing was performed at the facility level reported a similar testing rate (85.9%; 693/807) to our study.^11^

The difficulty of reaching children not living with their mothers requires addressing. Home testing may be an option but this approach has limitations because of frequent non-disclosure of the mother’s HIV status to her partner or family; fear that the child will test HIV-positive; and stigma.^29^ Other studies in Africa have assessed whether community and home-based testing could improve HIV testing in children. A study in Zimbabwe offering the choice between the two places for testing reported HIV testing uptake was only 60% (3638/6062) and significantly higher with home-based testing.^17^ However, in Kenya, most parents said they preferred clinic-based testing (76.6%; 377/492) to home-based testing.^30^ Home testing was feasible in eastern Africa and offered flexible options to parents, but this approach requires considerable resources for a relatively poor added value in terms of HIV testing. In addition, home-based testing may be more challenging in Burkina Faso because of the high level of stigma in having an HIV-infected child.^29^Importantly, our findings confirm that facility-based testing allows ART to be started quickly as recommended by WHO.^31^ All children diagnosed with HIV in our study started ART shortly after the HIV diagnosis.

Travel time, high costs^17^ and low educational level^10^^,^^32^ may be barriers to facility-based testing. Our approach, nested within the mother’s routine HIV care visit, eliminates these difficulties. Of note, having community health workers and health-care providers explain the course of HIV and the importance of child testing likely contributed to our high acceptance rate. 

As expected, the characteristics of the children infected with HIV and their mothers revealed a certain risk profile: younger age of mother; shorter period since mother started ART; poorer adherence to ART by the mother; and less awareness of the 18-month test. This last finding highlights the need to inform mothers of this final HIV test as part of the PMTCT programme. In addition, breastfeeding duration was longer in HIV-infected children, suggesting that some infections may have occurred after 12–18 months, as reported previously in Burkina Faso and other countries.^25^ A final HIV test for all children exposed to the virus whenever their exposure ends, for example when they stop breastfeeding whatever the age, is therefore vital.

The average cost for testing children seems feasible for the national AIDS control programme (US$ 2.5) and is lower than provider-initiated testing and counselling in South Africa (US$ 4.7).^33^

Our study has several limitations. Some HIV-infected children identified may have been previously reported by their mothers. We believe this bias was unlikely as we gave no incentive to the mother to have her child tested. In addition, we did not achieve the calculated sample size because the number of women with an eligible child was lower than we expected, which may have reduced the precision of our estimates. Finally, we did the cost assessment retrospectively as we did not originally plan to do one. However, we had precise records of costs so we believe our estimates are accurate.

To conclude, identifying undiagnosed children with HIV through their mothers’ routine follow-up in HIV outpatient clinics was feasible and effective in a typical west African context. Importantly, testing in a health facility enabled rapid initiation of ART for all HIV-infected children. This simple life-saving strategy, which both staff and mothers found acceptable, should be widely implemented.
